# mTOR as a Key Regulator in Maintaining Skeletal Muscle Mass

**DOI:** 10.3389/fphys.2017.00788

**Published:** 2017-10-17

**Authors:** Mee-Sup Yoon

**Affiliations:** Department of Molecular Medicine, School of Medicine, Gachon University, Incheon, South Korea

**Keywords:** mTOR, skeletal muscle, hypertrophy, atrophy, sarcopenia

## Abstract

Maintenance of skeletal muscle mass is regulated by the balance between anabolic and catabolic processes. Mammalian target of rapamycin (mTOR) is an evolutionarily conserved serine/threonine kinase, and is known to play vital roles in protein synthesis. Recent findings have continued to refine our understanding of the function of mTOR in maintaining skeletal muscle mass. mTOR controls the anabolic and catabolic signaling of skeletal muscle mass, resulting in the modulation of muscle hypertrophy and muscle wastage. This review will highlight the fundamental role of mTOR in skeletal muscle growth by summarizing the phenotype of skeletal-specific mTOR deficiency. In addition, the evidence that mTOR is a dual regulator of anabolism and catabolism in skeletal muscle mass will be discussed. A full understanding of mTOR signaling in the maintenance of skeletal muscle mass could help to develop mTOR-targeted therapeutics to prevent muscle wasting.

## Introduction

Skeletal muscle primarily functions as a motor for locomotion, and recently growing evidence has recognized skeletal muscle as a crucial regulator of whole body metabolism (Izumiya et al., 2008; McCarthy and Esser, 2010). Skeletal muscle mass is dependent on diverse conditions, including aging, disuse, cachexia, denervation, and burns (Glass, 2003), and affects disability, loss of independence, and increased risk of morbidity and mortality (Hornberger, 2011). Hence, the maintenance of muscle mass has been recognized as a determinant which directly influences quality of life.

Mechanical overload and anabolic stimulation are suggested to be important for increasing skeletal muscle mass and fiber size. Notably, changes in adult muscle mass by external stimuli emerged from the growth of the individual muscle fibers, not from an increase in the number of muscle fibers (Glass, 2005). One of the most widely recognized major players in controlling muscle mass is mammalian target of rapamycin (mTOR). mTOR is a serine/threonine kinase which senses various environmental and intracellular changes including nutrient availability and energy status, and coordinates diverse cellular processes including cell growth, differentiation, autophagy, survival, and metabolism (Laplante and Sabatini, 2012). Two biochemically and functionally distinct mTOR complexes, mTORC1 and mTORC2, exist (Laplante and Sabatini, 2012). Both complexes have mTOR as their common catalytic subunit and each has unique components. mTORC1 consists of the regulatory associated protein of mTOR (raptor), the 40 kDa proline-rich Akt substrate (PRAS40), the DEP domain-containing mTOR-interacting protein (DEPTOR), and the mammalian lethal with SEC13 protein 8 (mLST8). The other mTOR complex, mTORC2, shares mLST8 and DEPTOR with mTORC1 and includes unique components: the rapamycin-insensitive companion of mTOR (rictor), mammalian stress-activated map kinase-interacting protein 1 (mSIN1), and the exchange factor found in platelets and leukemia and neuronal tissues (Xpln). The rapamycin sensitive complex mTORC1 integrates several extracellular and intracellular signals including growth factors, amino acid availability, intracellular energy status, and oxygen levels. The tumor suppressor tuberous sclerosis complex TSC1-TSC2 mediates the upstream signals of mTORC1 except for amino acid availability by acting as a GTPase-activating protein (GAP) for the small GTPase Rheb. mTORC1 controls protein synthesis by activating S6 kinase 1 (S6K1) and inhibiting 4E-binding protein 1 (4EBP1) (Ma and Blenis, 2009). On the other hand, mTORC2 phosphorylates AGC kinases, serum, and glucocorticoid-regulated kinase 1 (SGK1), protein kinase C (PKC), and Akt, and regulates cell survival and metabolism (Sarbassov et al., 2005).

mTORC1 is known as a key regulator in controlling skeletal muscle mass following contraction and mechanical load-induced hypertrophy, synergistic ablation, myotube hypertrophy, and amino acid sensing, in which mTOR interacts with factors of both skeletal muscle hypertrophy and atrophy. This review explores the critical role of mTORC1 and its signaling in both catabolism and anabolism of skeletal muscle by summarizing genetic and pharmacological evidence and delineating the current understanding of the molecular mechanism of mTOR in the regulation of skeletal muscle mass.

## The phenotypes of muscle-specific deficiency of mTOR signaling

Understanding of the role of mTOR in muscle growth and hypertrophy has progressed recently from the evidence of several loss-of-function animal models, although it is relatively well-understood as being similar to the mechanism of the function of mTOR in cell growth regulation. To avoid the early embryonic mortality of mice deficient for mTOR and rictor/raptor, muscle-specific knockout mice of mTOR and mTOR components were generated (Guertin et al., 2006; Bentzinger et al., 2008).

Muscle-specific mTOR knockout mice (mTOR-) exhibit severe myopathy leading to premature death between 22 and 38 weeks of age (Risson et al., 2009). Muscle-specific raptor knockout mice (RAmKO) and raptor and rictor double-knockout mice (DmKO) show myopathy similar to one associated with the loss of mTOR in muscle, whereas muscle-specific rictor knockout mice (RImKO) do not. The deficiency of either mTOR or raptor reduces the phosphorylation of mTORC1 downstream targets, such as p70S6K1 and 4EBP1 and increases the phosphorylation of Akt at S473 and T308. Unexpectedly, Akt phosphorylation in DmKO is comparable to phosphorylation in RAmKO, implying that mTORC2 is not required for Akt activation in muscle. In addition, the muscle of RImKO mice is similar to one of wild type controls, suggesting that mTORC1 plays a major role in the metabolic processes and functions of muscle.

Compared to RAmKO/DmKO, mTOR- mice shows changes in muscle contractile properties. Additionally, mTOR- reduces the transcription of dystrophin, resulting in a decrease in the content of the dystrophin-glycoprotein complex (DGC), which connects the cytoskeleton of a muscle fiber to its surrounding extracellular matrix; the disruption of DGC results in muscular dystrophy. mTOR directly binds to the promoter of the dystrophin gene to control the transcription of dystrophin in a cell-autonomous, rapamycin-resistant, and kinase-independent manner. mTOR knockout muscle also undergoes metabolic changes, resulting in glycogen accumulation due to increased glycogen synthesis and glucose uptake together with reduced glycogen breakdown through glycogenolysis and the glycolytic and oxidative pathways. mTORC1 deficiency in muscle significantly reduces the expression of genes in mitochondria biogenesis, such as proliferator-activated receptor γ coactivator-1 alpha (PGC1α), myoglobin, PPARγ, and cytochrome C oxidase IV (COXIV). However, it does not affect either intramuscular ATP level or whole body glucose homeostasis.

Similar to mTOR deficiency in muscle, RAmKO induces progressive dystrophy, impairment in oxidative capacity, and increased glycogen stores; these mice displayed metabolically fast-twitch, glycolytic skeletal muscle (Bentzinger et al., 2008). This characterization is also reflected in the overall metabolism, which displayed lower glucose uptake from the blood. However, the soleus and EDL of RAmKO muscle had slower myosin heavy chain (slMHC)-positive fibers, indicating that RAmKO muscle contained more structurally slow-twitch, oxidative skeletal muscle fibers. In addition, the deletion of S6K1, an mTORC1 downstream target, in muscle increases AMP/ATP level and activates AMPK, resulting in energy stress and muscle cell atrophy (Aguilar et al., 2007).

Even though the phenotypes of mTORC1-signaling deficient mice are similar in terms of skeletal muscle myopathies, mitochondrial and oxidative metabolism in these mice are distinct. The deficiency of mTOR and raptor in muscle induces defects in mitochondrial metabolism and a decrease in mitochondrial gene expression (Bentzinger et al., 2008; Risson et al., 2009). This is supported by previous reports that mTORC1 is a positive regulator of PGC1α, a master regulator of mitochondrial biogenesis. However, S6K1 -/- skeletal muscle has high mitochondrial content accompanied by increased expression of mitochondrial genes, which protect against diet-induced obesity together with enhanced β-oxidation in white adipose tissue (WAT) (Um et al., 2004). These observations imply that an mTORC-mediated regulation is vital for mitochondrial metabolism in metabolism-related organ, which is differentially regulated in muscle and WAT, respectively. This differential regulation of mitochondrial metabolism warrants further investigation.

## mTOR signaling regulates muscle protein synthesis

### The role of mTOR in IGF-I dependent pathway of skeletal muscle

Numerous reports show that IGF-I is a requisite for muscle growth and regeneration (Florini et al., 1991; Vandenburgh et al., 1991; Coleman et al., 1995; Musaro et al., 2001; Rabinovsky et al., 2003; Pelosi et al., 2007), as well as a well-known upstream stimulator of mTOR in skeletal muscle. IGF-I binds to IGF-I receptor (IGFR), a receptor tyrosine kinase, and subsequently recruits insulin receptor substrate-1 (IRS-1). Although IRS-1 activates the Ras-Raf-MEK-ERK pathway, the role of this pathway in skeletal muscle is not clear (Rommel et al., 1999). Instead, Akt /mTOR signaling by IGF-I/IGFR/IRS-1 has been shown to be indispensable in prompting muscle hypertrophy (Glass, 2003). Akt phosphorylates TSC1/2, which inhibits the GTPase-activating protein (GAP) activity of TSC1/2 toward small G protein Rheb. Then, GTP-bound Rheb activates mTORC1, resulting in phosphorylation of S6K1and 4EBP1, which promote protein synthesis by activating ribosomal protein S6 and by releasing the translation initiation factor eIF-4E, respectively. In line with IGF-I-Akt-mTORC1 regulation, IGF-I induces hypertrophy of skeletal myofiber in tissue culture (Vandenburgh et al., 1991). Muscle-specific expression of IGF-I in transgenic mice results in at least a 2-fold increase in muscle hypertrophy (Coleman et al., 1995; Musaro et al., 2001), suggesting that the IGF-I/Akt/mTORC1 pathway is indispensable to muscle hypertrophy. In addition, Akt regulates muscle mass by phosphorylating and deactivating glycogen synthase kinase (GSK) β1, followed by the GSK β1-dependent inhibition of the eukaryotic translation initiation factor 2B (eIF2B) (Manning and Cantley, 2007; Schiaffino and Mammucari, 2011).

However, a recent report showed that IGF-I and its receptor IGFR were not important to the induction of hypertrophy and the activation of Akt/mTOR in mechanical loading (Spangenburg et al., 2008). The expression of dominant negative (DN)-IGF-I receptor specifically in skeletal muscle induced muscle hypertrophy using an increased functional overload model induced by synergistic ablation (Spangenburg et al., 2008). Of interest, DN-IGF-I receptor-expressing muscle showed a similar level of activation of Akt and p70S6K1. These results implied that an unknown upstream mediator beyond IGFR might regulate Akt/mTOR signaling in skeletal muscle hypertrophy.

### The role of mTOR in IGF-I independent pathway of skeletal muscle; PA-induced mTOR activation in mechanical stimulus

One of the potential IGFR-independent mTOR regulators in skeletal muscle is phosphatidic acid (PA). Hornberger et al. observed that IGF-I-independent mechanical stretch increases phosphatidic acids (PA), followed by mTOR activation (Hornberger et al., 2006). PA directly binds to the FKBP12-rapamycin binding (FRB) domain in competition with rapamycin, and activates mTOR (Fang et al., 2001). PA is synthesized through several pathways: from phosphatidylcholine (PC) by phospholipase D (PLD), from lysophosphatidic acid (LPA) by lysophosphatidic acid acyltransferases (LPAAT), and from diacylglycerol (DAG) by diacylglycerol kinase (DGK) (Wang et al., 2006; Yoon et al., 2015). Among the several enzymes involved in PA biogenesis, PLD activity was increased by mechanical stretch and followed by mTOR activation (Hornberger et al., 2006). In addition, treatment with 1-butanol, a PLD inhibitor, inhibited the increase in mTOR activity, supporting the role of PLD in mechanical stretch (Hornberger et al., 2006). However, PA level continued to remain high after the elevated PLD activity returned to basal level 15 min after mechanical stretch using [3H] arachidonic acid labeling, suggesting that other enzymes produce PA under mechanical stretch. Hornberger et al. found that DGK ζ produces PA under mechanical stimulation, which is followed by mTOR activation (You et al., 2014). Mechanical stimulation does not induce PLD activity under [3H] myristic acid labeling that preferentially labels PC, and FIPI, a PLD inhibitor, did not inhibit mechanical stimulation. Instead, both DAG and membrane DGK activity, which are critical for mTOR activation, were increased during mechanical stimulation. However, the previous report suggested that PLD1-produced PAs preferentially bind to the FRB domain of mTOR and displace DEPTOR, leading to mTORC1 activation (Yoon et al., 2015). Hence, further investigation into whether PA produced by DGK ζ during muscle stretch binds to the FRB domain or activates mTOR through an FRB-independent mechanism is warranted.

### The role of mTOR in IGF-I independent pathway of skeletal muscle; mechanism of mTORC1 activation by mechanical stimulus

It has been established that mTORC1 translocates to the lysosome through regulation of Ragulator-Rag in amino acid signaling (Sancak et al., 2008, 2010). Lysosomal localization of mTOR does not activate mTOR directly, but rather provides close proximity to Rheb, an essential activator of mTOR (Saxton and Sabatini, 2017). mTORC1 is activated by direct interaction with the GTP-bound form of Rheb (Sancak et al., 2008, 2010), which is regulated by the TSC complex [TSC1, TSC2, and Tre2-Bub2-Cdc16-1 domain family member 7 (TBC1D7) Dibble and Manning, 2013], a GAP of Rheb (Huang and Manning, 2008; Saxton and Sabatini, 2017). The location of Rheb, shown to be on the lysosome, was not changed by either amino acids or insulin (Menon et al., 2014). Nevertheless, the TSC complex activates the intrinsic GTPase activity of Rheb on the surface of the lysosome and localizes to the lysosome, at least partially through its association with Rheb-GDP in the absence of growth factors (Menon et al., 2014). Insulin activates Akt, which subsequently phosphorylates the TSC complex, resulting in the dissociation of the TSC complex from Rheb, followed by Rheb GTP loading and mTORC1 activation (Menon et al., 2014).

The Hornberger group found that mTOR and TSC2 were highly enriched in the lysosome of the muscle in the resting state (Jacobs et al., 2013). Mechanical stimulation induces TSC2 phosphorylation at RxRxxS^*^/T^*^, which resulted in the dissociation of TSC2 from the lysosome and the subsequent change of Rheb to the active Rheb-GTP state. Furthermore, mechanical stimulation also facilitates the association of mTOR with the lysosome. Accordingly, mTOR potentiates the activation of the lysosome through the interactions with Rheb-GTP or PA, as previously reported (Sancak et al., 2008, 2010; Yoon et al., 2011). However, the kinase for TSC phosphorylation was unclear in this study since mechanical stimulation was previously shown to activate mTOR in PI3K/Akt-independent manner (Hornberger et al., 2004; O'Neil et al., 2009). In addition, Song et al. recently suggested the colocalization of mTOR with eukaryotic translation initiation factor 3 subunit F (eIF3F) in resistance exercise (Song et al., 2017). mTOR is localized on the lysosome in the basal state and the mTOR-LAMP2 complex is translocated to the cell periphery under resistance exercise, which provide close proximity to the capillaries. In support of this, the lysosome is shown to migrate to the cell periphery after nutrient stimulation through two kinetin proteins, K1F1Bβ and KIF2, which are essential to mTORC1 activation (Korolchuk et al., 2011). Concurrently, TSC2 dissociates from Rheb, followed by the reduction of TSC2 on the cell periphery and the subsequent increase of mTORC1 activity (Song et al., 2017). In addition, both the association of mTOR with eIF3F and S6K1 activity are increased in fed conditions after exercise, which provides an explanation for the enhanced muscle protein synthesis (Song et al., 2017). Nevertheless, the association of mTOR with eIF3F was mainly determined by using an immunofluorescent approach in Song's study, which requires further investigation by using a range of techniques.

## mTOR regulation signals muscle wasting

The loss of skeletal muscle, muscle atrophy, stems from an increase in the rate of protein degradation or the decrease of protein synthesis under various conditions, such as disuse, diseases, and aging. In line with mTOR function as a positive regulator of muscle hypertrophy, mTOR signaling is negatively regulated by muscle atrophy-inducing signals or blocks muscle atrophy signals. In this section, it will be discussed the crosstalk between mTOR and two major muscle atrophy-inducing signals such as myostatin and glucocorticoids.

### The crosstalk between mTOR and myostatin (growth differentiation factor 8, GDF-8)

Myostatin, a transforming growth factor-β (TGF-β) family member, plays a critical role in inhibiting the growth of muscle mass and muscle cell differentiation (McPherron et al., 1997). The deletion of myostatin in mice results in muscle hyperplasia and hypertrophy, and more than doubles skeletal muscle (McPherron et al., 1997). Myostatin regulates the number of muscle fibers during development and the growth of muscle fibers postnatally (Lee, 2007). The binding of myostatin to the type II activin receptor IIb leads to interaction with the type I receptor ALK4 or ALK5, which results in the phosphorylation and activation of the transcription factors Smad2 and Smad3(Sartori et al., 2014). Additionally, myostatin decreases Akt phosphorylation, which is accompanied by the accumulation of dephosphorylated active Forkhead Box-O1 (FOXO1) and FOXO3, followed by upregulation of components of the ubiquitin-proteasome pathway, such as atrogin-1 and the muscle-specific E3 ubiquitin ligase muscle RING-finger1 (MURF1) (McFarlane et al., 2006; Lokireddy et al., 2011). In addition, myostatin blocks differentiation-inducing genes, such as myogenin and myoD (Trendelenburg et al., 2009), suggesting that myostatin regulates muscle differentiation by modulating both the programs of differentiation and atrophy.

mTOR regulation by myostatin has sophisticated the molecular mechanism of myostatin signaling. The overexpression of myostatin decreases Akt and mTORC1 components, such as p70S6K1, S6, and 4EBP1 (Amirouche et al., 2009). Supporting the negative regulation of myostatin in mTORC1 signaling, genetic deletion of myostatin elevates the activities and the expression levels of Akt, p70S6K1, and S6 (Lipina et al., 2010). Additionally, treatment with myostatin reduces myoblast differentiation and myotube size by inhibiting the activity of Akt/mTORC1/p70S6K1 in human skeletal muscle cells (HuSkMC) (Trendelenburg et al., 2009). The depletion of raptor increases myostatin-induced Smad2 phosphorylation, followed by further inhibition of myostatin-induced muscle differentiation. The knockdown of rictor itself inhibits muscle cell differentiation, and does not affect myostatin-induced pSmad2 and muscle differentiation. These results suggested that both mTORC1 and myostatin-Smad2 signaling negatively regulate each other. The overexpression of ActRIIB induces inhibition of myostatin, resulting in skeletal muscle hypertrophy, which is reduced partially by treatment with rapamycin (Sartori et al., 2009). Hence, the studies suggested that myostatin attenuates protein synthesis in muscle by coordinating the crosstalk between myostatin-mediated and mTOR signaling. However, on the other hand, several studies suggested that mTOR signaling and myostatin signaling could separately regulate muscle growth. The injection of a myostatin antibody enhances phosphorylation of p70S6K1 and S6 in muscle, but does not change phosphorylation of Akt and 4EBP1 in the concomitant increase of myofibrillar synthesis (Welle et al., 2009). In this study, treatment with rapamycin does not affect myofibrillar synthesis, while it decreases the phosphorylation of p70S6K1 and S6, implying that mTOR is not involved in myostatin-mediated myofibrillar synthesis (Welle et al., 2009). In addition, follistatin, an inhibitor of myostatin, activates Akt/mTOR/p70S6K1/S6 signaling in muscle growth, which exists independently of myostatin-driven mechanisms (Winbanks et al., 2012), supporting the disconnection between myostatin and mTOR signaling. Hence, myostatin may regulate protein synthesis in both an mTOR-dependent and an mTOR-independent manner; it controls the translation through Akt/mTORC1/p70S6K1/S6 signaling and, at the same time, it directly acts on unknown regulators of translation.

### The role of mTOR in glucocorticoid-induced atrophy

Glucocorticoids are some of the most fundamental regulators of energy homeostasis and adjust the metabolism of carbohydrates, fat, and protein in skeletal muscle (Munck et al., 1984). Glucocorticoids bind to the glucocorticoid receptor (GR), which translocates to the nucleus and binds to the glucocorticoid response element (GRE) in the promoters of target genes (Meijsing et al., 2009). Notably, the circulating levels of glucocorticoids are increased under many pathological conditions which are accompanied by muscle atrophy such as cachexia, starvation, sepsis, metabolic acidosis, and severe insulinopenia (Braun and Marks, 2015). Exogenous administration of glucocorticoids induces muscle atrophy and the blockage of GR; adrenalectomy or treatment with the GR antagonist RU486 diminishes muscle atrophy in sepsis, cachexia, starvation, and severe insulinopenia (Menconi et al., 2007; Schakman et al., 2008). Hence, endogenous glucocorticoids are critical regulators in muscle atrophy.

The crosstalk between GR and mTOR has been reported in muscle cells (Shimizu et al., 2011). Tanaka et al. found that REDD1 and KLF15 inhibit mTOR activation as direct targets of GR. REDD1 has a functional GRE, which sequestrates 14-3-3 from TSC1/2, resulting in activation of mTOR (DeYoung et al., 2008). KlF15 plays a critical role in muscle catabolism through the transcriptional upregulation of atrogen-1, MuRF-1, and branched-chain aminotransferase 2 (BCAT2). BCAT2 catalyzes the first reaction of BCAA catabolism, facilitating BCAA degradation, followed by mTOR inactivation and decrease of myofiber size (Shimizu et al., 2011). On the other hand, mTOR negatively modulates GR-mediated transcription by inhibiting GR recruitment of target genes. Hence, both GR and mTOR control each other exclusively in the regulation of muscle mass.

Glucocorticoids also elicit muscle atrophy via controlling transcription of myostatin, an inhibitory regulator of muscle growth, which we discussed in the previous section. Human myostatin promoter is reported to have a putative GRE and is responsive to dexamethasone and RU-486, an antagonist of GR (Ma et al., 2001). Indeed, the expression of myostatin mRNA and protein are increased in a dose-dependent manner in dexamethasone-treated rats (Ma et al., 2003). Myostatin production is also induced by food deprivation in a glucocorticoid-dependent manner (Allen et al., 2010). A recent report also suggests that glucocorticoids increase phosphorylation of CEBP by decreasing PDE3/4 and activating PKA through inhibiting Akt activity, resulting in increased myostatin expression (Xie et al., 2017). Hence, glucocorticoids may regulate mTOR by modulating the level of both BCAT2 and myostatin to regulate catabolism in skeletal muscle.

## mTOR and sarcopenia

Sarcopenia has been defined as an age-related continuous decline in muscle mass, quality, and strength (Sakuma et al., 2014). It is characterized by overall decreases in size and number of skeletal muscle fibers, mostly the type 2 or fast-twitch muscle fibers, and a marked infiltration of fibrous and adipose tissue into the skeletal muscle (Walston, 2012). The amount of circulatory IGF-I and IGF-I mRNA levels are reduced (Leger et al., 2008), and subsequently the activity of Akt/mTOR/p70S6K1 are decreased in older age groups compared to one in younger groups (Pallafacchina et al., 2002; Cuthbertson et al., 2005; Leger et al., 2008). A study using inducible liver IGF-I-deficient mice revealed that reduced IGF-I at 1 year of age is related to deteriorated health accompanied by age-related pathologies (Gong et al., 2014), consistent with the previous report that virus-mediated IGF-I gene transfer counteracts the decreases of muscle mass and force in aged groups(Barton-Davis et al., 1998). In addition, the levels of IGF-binding proteins in aged groups increased, followed by inactivation of Akt, impaired differentiation, and hypertrophy of myotubes (Deane et al., 2013; Sharples et al., 2013), suggesting that lowering of IGF-I signaling are responsible for the decrease of skeletal muscle mass in old-aged group. On the other hand, recent papers report contradicting results in IGF-I levels and activity of Akt/mTOR/p70S6K1 in aged muscles. IGF-1 mRNA expression was not changed or even increased in aged muscles compared to young muscles in humans (Sandri et al., 2013), whereas it was decreased in aged muscle of mice (Drummond et al., 2008; Sandri et al., 2013). Moreover, the correlation between IGF-1 level and Akt/mTOR was not consistent in several reports (Sandri et al., 2013; Markofski et al., 2015). Thus, the attempt to change IGF-I levels in aged muscle for sarcopenia warrants further investigations

Of note, the hyperphosphorylation of mTORC1 was observed in aged human muscles (Sandri et al., 2013; Markofski et al., 2015). Inhibition of mTORC1 has a positive effect in the animal model of age-associated muscular dystrophy (Ramos et al., 2012). Related to this, reduced mTOR signaling has been shown to regulate longevity in human and model organisms (Powers et al., 2006; Bjedov et al., 2010; Robida-Stubbs et al., 2012; Passtoors et al., 2013) and reduce age-related pathologies (Johnson et al., 2013a). Inhibition of mTOR signaling in aged muscle may have similar beneficial effects on multiple age related pathologies (Johnson et al., 2013b). Nevertheless, the hyperactivation of mTOR in aged muscles does not induce protein synthesis (Markofski et al., 2015). Chronic mTORC1 activation through TSC1 knockout in old muscle leads to muscle atrophy mainly due to inability to induce autophagy (Castets et al., 2013), suggesting the importance of mTOR-induced regulation of autophagy in aged muscle. Moreover, the hyperphosphorylation of mTOR might lead to resistance to anabolic stimuli in aged muscle. Anabolic stimuli such as muscle contraction, insulin, and nutrients cause an increase in protein synthesis through mTOR activation in the muscle, and this anabolic stimuli-induced mTOR activation is reduced in individuals belonging to older age groups compared to younger adults (Parkington et al., 1985; Cuthbertson et al., 2005; Chale-Rush et al., 2009; Timmerman et al., 2010; Fry et al., 2011; Li et al., 2012). Hence, the hypertrophic response by mTOR activation is important for overall muscle maintenance in aged muscle.

## Conclusions and perspectives

**Figure 1:**
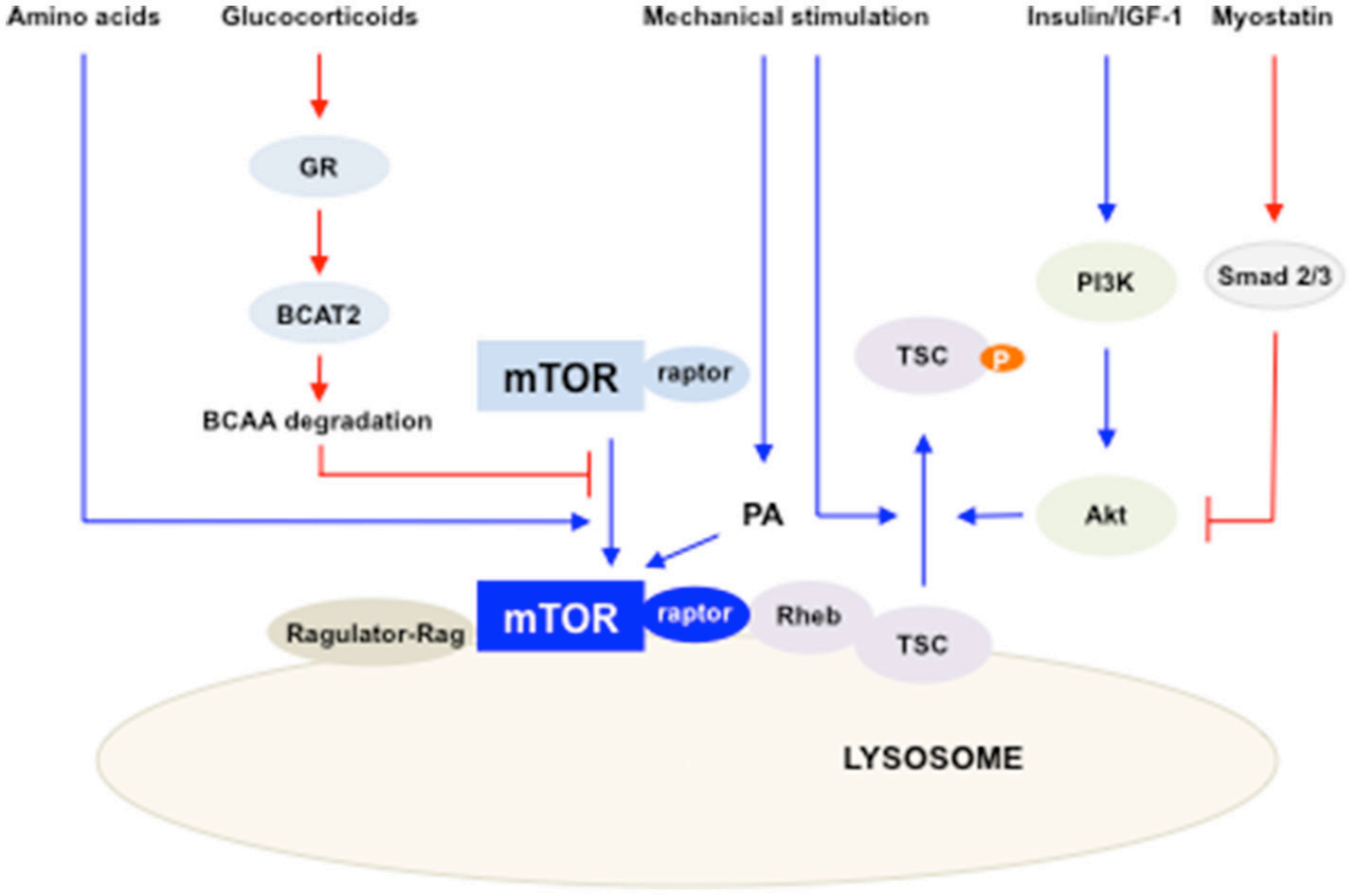
The summary of the regulation of mTORC1 activity in skeletal muscles. Multiple factors and pathways affect mTORC1 activity to regulate skeletal muscle mass. mTORC1 is activated by IGF-I/insulin, mechanical stimulation and amino acids (blue lines) and inhibited by glucocorticoids and myostatin (red lines). Activated mTORC1 increases protein synthesis in skeletal muscle.

Our understating of mTOR regulation in both skeletal muscle hypertrophy and atrophy has been advanced in recent years. While mTOR has been appreciated as a main regulator of protein synthesis in skeletal muscle, its crosstalk with muscle atrophy inducing triggers, such as myostatin and glucocorticoids, have been studied. The diverse involvements of mTOR in maintaining skeletal muscle mass have shed light on the complexity of the role of mTOR in skeletal muscle hypertrophy and atrophy (Figure 1). Hence, dissection of mTOR signaling provides useful potential therapeutic strategies in boosting skeletal muscle growth and preventing muscle loss.

Recent studies suggest an additional role of mTOR in skeletal muscle related to the regulation of non-coding RNAs. MicroRNAs (miRNAs) are small non-coding RNAs that are ~21–23 nucleotides in length, which bind to 3′-UTR of target mRNAs and function in gene silencing and translational suppression (Zhang et al., 2016). Several miRNAs are identified as myomiRNAs, which are enriched in skeletal muscle and known to modulate the cellular processes involved in muscle growth, development, and maintenance, including hypertrophy and atrophy. The expression of several miRNAs, such as miR-1, miR-133, miR-206, and miR-125b, are regulated by mTOR directly or indirectly (Sun et al., 2010; Ge et al., 2011), suggesting the additional regulation of mTOR in skeletal muscle mass. Recently, a novel polypeptide encoded by the long non-coding RNA (lncRNA) LINC00961 was shown to regulate mTOR activation and muscle regeneration (Matsumoto et al., 2017), implying crosstalk between mTOR and non-coding RNAs in skeletal muscle. In this context, future studies of mTOR signaling as a possible therapeutic target using non-coding RNAs are warranted.

## Author contribution

The author confirms being the sole contributor of this work and approved it for publication.

### Conflict of interest statement

The authors declare that the research was conducted in the absence of any commercial or financial relationships that could be construed as a potential conflict of interest.
